# *Guizhi-Fuling-Wan*, a Traditional Chinese Herbal Medicine, Ameliorates Memory Deficits and Neuronal Apoptosis in the Streptozotocin-Induced Hyperglycemic Rodents via the Decrease of Bax/Bcl2 Ratio and Caspase-3 Expression

**DOI:** 10.1155/2012/656150

**Published:** 2012-10-31

**Authors:** Kuo-Jen Wu, Yuh-Fung Chen, Huei-Yann Tsai, Chi-Rei Wu, W. Gibson Wood

**Affiliations:** ^1^Department of Chinese Pharmaceutical Sciences and Chinese Medicine Resources, College of Pharmacy, China Medical University, Taichung 40402, Taiwan; ^2^Department of Pharmacology, College of Medicine, China Medical University, Taichung 40402, Taiwan; ^3^Department of Pharmacy, China Medical University Hospital, Taichung 40421, Taiwan; ^4^Department of Pharmacology, University of Minnesota and Geriatric Research, Education and Clinical Center, VA Medical Center, Minneapolis, MN 55455, USA

## Abstract

Brain neuronal apoptosis and cognitive impairment are associated with hyperglycemia and diabetes mellitus. The present study determined if the Chinese herbal medicine *Guizhi-Fuling-Wan* (GFW) would reduce memory loss and neuronal apoptosis in streptozotocin- (STZ-) induced hyperglycemic rodents. Two weeks after STZ induction, GFW was orally administered once daily for 7 days. GFW significantly improved spatial memory deficits in STZ-induced hyperglycemic mice. GFW decreased TUNEL-positive cells and caspase-3 positive cells in STZ-induced hyperglycemic rats. It also was found that GFW treatment reduced caspase-3 protein levels and increased levels of the antiapoptotic protein Bcl-2 that were indicative of neuroprotection. The protective therapeutic effects of GFW on neuronal apoptosis and cognition deficits caused by STZ-induced hyperglycemia may be due in part to inhibition of the cellular apoptosis pathway. GFW may have therapeutic effects in patients with diabetes-mellitus-induced neuropathology.

## 1. Introduction

Diabetes mellitus is an increasingly common metabolic disorder characterized by hyperglycemia, frequent urge to urinate (polyuria), increased thirst (polydipsia), increased hunger (polyphagia), and weight loss. Hyperglycemia is often associated with complications such as peripheral and autonomic neuropathy [1]. Diabetes has been shown recently to have effects on the central nervous system in the hippocampus and cortex [2]. The hippocampus is a critical integration center for cognitive functions [3]. The cortex plays a prominent role in working memory and executive function [4]. Diabetes is associated with apoptosis [5] which could be a mechanism for hyperglycemia-induced neuronal cell death [2]. Hyperglycemia induced by streptozotocin stimulates apoptotic pathways [6]. Apoptosis occurs in several neurodegenerative disorders like ischemic stroke, Alzheimer's disease, and Parkinson's disease [7]. The process of apoptosis includes condensation of chromatin, shrinkage of the cell and nucleus, membrane blebbing, and DNA fragmentation [8]. Two main families of proteins, caspase enzymes and Bcl-2 family members are, the key elements in apoptosis. In the caspase family, caspase-3 plays a pivotal role in apoptosis [9]. For the Bcl-2 family, there are the antiapoptotic proteins including Bcl-2 and Bcl-xL and proapoptotic proteins including Bax and Bak [10].

Guizhi-Fuling-Wan (abbreviated as GFW), a traditional Chinese herbal medicine, consists of five herbal components: *Cinnamomum cassia* Blume, *Poria cocos *(Schw.) Wolf, *Paeonia lactiflora* Pall., *Paeonia suffruticosa* Andr., and *Prunus persica* (L.) Batsch. GFW has been used mainly to treat gynecological diseases for thousands of years. GFW is protective in various experimental conditions including the reduction of endometrial explants [11] and invasion of human cervical cancer cells [12]. GFW was neuroprotective in models of cerebral ischemia and diabetes. Nitric oxide donor-induced neuronal cell death was reduced by GFW and attributed to inhibition of apoptosis [13]. The purpose of the present study was to determine if GFW would reduce memory deficits and neuronal apoptosis in STZ-induced hyperglycemic rodents.

## 2. Materials and Methods

### 2.1. Reagents

Acetic acid, *tert*-butylhydroquinone(BHQ), cinnamaldehyde, cinnamic acid, gallic acid, paeoniflorin, paeonol, and streptozotocin (STZ) were from Sigma-Aldrich (St. Louis, MO, USA). Zoletil was from Virbac Laboratories (Carros, France). BCA protein assay kit was from Thermo Fisher Scientific (Lafayette, CO, USA). NovoLink Ploymer Detection System Kit was from Leica Microsystems Inc. (Newcastle Upon Tyne, UK). Apo-BrdU-IHC *in situ *DNA Fragmentation Assay Kit was from BioVision (Milpitas, CA, USA). Anti-caspase-3 antibody was from GeneTex Inc (Irvine, CA, USA). Anti-Bcl-2 antibody, anti-Bax antibody, and anti-*β*-actin antibody were from Santa Cruz Biotechnology (Santa Cruz, CA, USA).

### 2.2. Extraction of GFW and Determination of Chemical Compounds by HPLC

**Figure 1:**
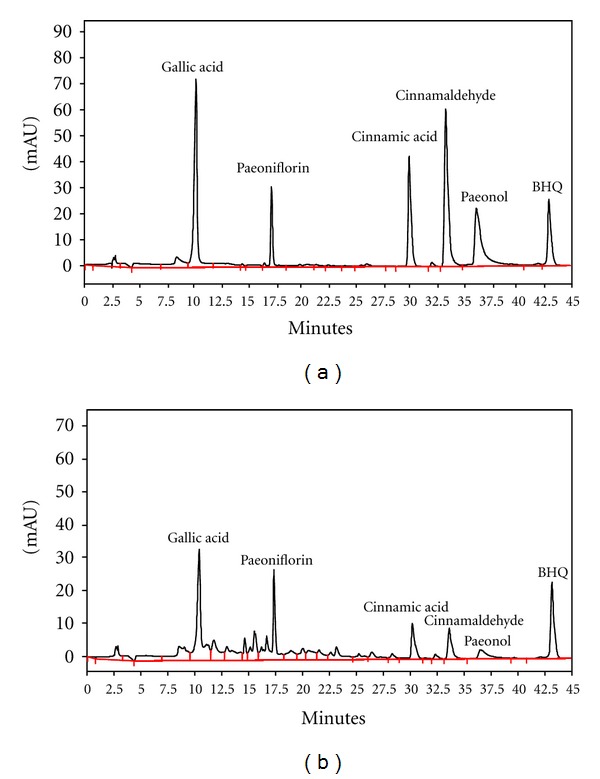
HPLC chromatograms of GFW at 250 nm. Trace: (a) standard, (b) GFW. BHQ: *tert*-butylhydroquinone as an internal standard.

GFW consists of five medicinal herbs at a ratio of 1 : 1 : 1 : 1 : 1, including* Cinnamomum cassia* Blume, *Poria cocos *(Schw.) Wolf, *Paeonia lactiflora* Pall, *Paeonia suffruticosa* Andr, and *Prunus persica* (L) Batsch. Each medicinal plant was 200 g and extracted twice with 2 L boiling distilled water for 2 hr. The extracts were filtered and freeze dried. The yield ratio of GFW extracts was 11.54%. GFW was freshly prepared in distilled water prior to administering to animals. HPLC-DAD was used to analyze GFW components using procedures reported previously [14]. Briefly, GFW was dissolved in methanol and then filtered with a 0.22 *μ*m membrane filter (Millipore, MA, USA). Stock solutions of standards were prepared to final concentrations of 2.5 mg/mL in methanol. All standards and sample solutions (20 *μ*L) were injected in triplicate. A Shimadzu VP series HPLC system and Shimadzu Class-VP chromatography data system were used. All chromatographic operations were carried out at 25°C. The chromatographic peaks of compounds were confirmed by comparing their retention times and UV spectra. A LiChrospher RP-18e (250 × 4 mm, 5 *μ*m) column (Merck KGaA, Darmstadt, Germany) was used. Chromatographic separation of compounds, including gallic acid, paeoniflorin, cinnamic acid, cinnamaldehyde, and paeonol, was carried out using a two-solvent system. Solvent A was 100% acetonitrile and solvent B was 0.2% acetic acid at pH = 3.23. The analyses were performed by a gradient program. The conditions were as follows: initial condition of 100% solvent B, 0–10 min changed to 80% solvent B, 10–30 min changed to 60% solvent B, and 30–45 min changed to 40% solvent B. Signals were detected at 250 nm. Tert-butylhydroquinone (BHQ, 25 *μ*g/mL) was used as an internal standard. HPLC chromatograms of standards and GFW are shown in Figure 1.

### 2.3. Animals and Drug Administration

**Figure 2:**
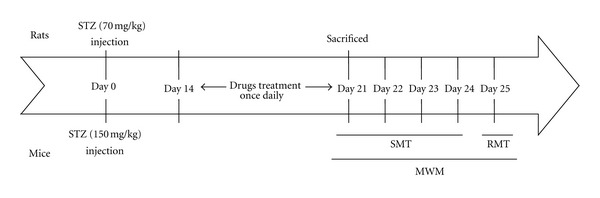
Schedule of drug treatment and experiment orders. GFW was administrated orally daily for 7 days and 1 h prior to MWM. On days 21–24 after STZ injection in mice, the spatial memory test (SMT) of the Morris water maze (MWM) was performed 4 trials a day for 4 consecutive days, followed 24 h later (day 25) by the reference memory test (RMT). On day 21, rats were sacrificed after being administrated orally GFW 1 h later.

Male ICR mice, weighing 20–22 g, and male Sprague-Dawley (SD) rats, weighing 225–275 g, were purchased from BioLASCO Co. Ltd. (Taipei City, Taiwan). All animals were fed with normal chow and housed in standard cages at a constant temperature of 22 ± 1°C relative humidity 55 ± 5% with 12 hr inverted light-dark cycle for at least 1 week at least before treatments. The experimental protocol was approved by the Committee on Animal Research, China Medical University and protocol number 99-12. The minimum number of animals and duration of observations required to obtain consistent data were used. The mice (*n* = 40) and the rats (*n* = 60) were randomly assigned to 5 groups: normal group, the hyperglycemia group, the GFW (1.0, 2.0 and 4.0 g/kg)-treated hyperglycemia group (each *n* = 8 in mice and *n* = 12 in rats). Hyperglycemia was induced by intraperitoneal injection of streptozocin (STZ) (Sigma, St. Louis, MO) at 150 mg/kg in mice and 70 mg/kg in rats [15]. Three days after STZ injection and overnight fasting, blood glucose was sampled from the tail vein and determined by using an automatic glucometer (ACCU-CHEK Active, Roche Diagnostics Ltd, Mannheim, Germany). Animals with a plasma glucose level higher than 300 mg/dL were considered hyperglycemic. The day of STZ injection was designated as day 0. Fourteen days after STZ injection, GFW was orally administered once daily for 7 days. Twenty-one days after STZ injection, rats were sacrificed for biochemical assays, and mice were used in behavioral test. The treatment schedule is shown in Figure 2.

### 2.4. Morris Water Maze Test

Behavioral testing was performed in a water maze [16, 17] which consisted of a circular stainless pool (90 cm in diameter; 60 cm in height) with a black-painted inner surface. The pool was filled with water to a depth of 35 cm (maintained at 23.0 ± 1.0°C). A circular transparent platform (10 cm) was submerged 1 cm below the water level and located in a constant position in the middle of one quadrant, equidistant from the center and edge of the pool. For each training session, the mice were put into the water at one of three starting positions, the sequence of the positions being selected randomly. In each training session, the latency to escape onto the hidden platform was recorded with a camera fixed on the ceiling of the room and stored in a computer. 

In the hidden-platform test, the mice were given 3 trials per day. Training was conducted for 4 consecutive days. During each trial, the mice were released from three pseudorandomly assigned starting points and allowed to swim for 60 s. The animals were allowed to remain there for 30 s after mounting the platform, and they were then placed in the home cage until the start of the next trial. The mouse was guided to the platform and allowed to rest on the platform for 30 s when it was unable to find the platform within 60 s. In the probe trial, the hidden platform was removed, and the animal was allowed to swim freely for 60 s. The parameter measured during the probe trial was the time spent in the quadrant of the target platform.

### 2.5. TUNEL Assay

Twenty-one days after STZ injection, rats were deeply anesthetized by intraperitoneal injection of 50 mg/kg of zoletil; intracardiac perfusion with 200 mL of 0.9% saline, followed by 4% paraformaldehyde in 0.1 M PBS, was performed before animals were decapitated. The brains were removed and then immersed in 10% paraformaldehyde, sectioned at 2 mm intervals using a rodent brain matrix slicer (RBM-4000C; ASI Instruments, Warren, MI, USA), processed and embedded in paraffin, then cut 2.5 *μ*m thick on a microtome, mounted on glass slides, and processed for immunohistochemical staining and TUNEL staining. The TUNEL assay was utilized on the brain sections using Apo-BrdU-IHC *in situ *DNA Fragmentation Assay Kit (BioVision, Milpitas, CA, USA). Brain slices were incubated with proteinase K for 20 min followed by 3% H_2_O_2_ in methanol for 5 min to inactivate endogenous peroxidase. TdT was added at room temperature and incubated overnight. A dark brown color indicating DNA breaks developed after incubation with DAB (3,3′-diamonobenzidine tetrachloride) and hydrogen peroxide, followed by counterstaining with methyl green. The percentages of positive TUNEL staining cells within areas of the cortex and hippocampus were estimated.

### 2.6. Immunohistochemical Staining

Brain slices were incubated with anti-caspase-3 antibody (GTX73090, dilution 1 : 200, GeneTex Inc., USA) overnight and immunohistochemical labeled using a NovoLink Polymer Detection System Kit (Leica Microsystems Inc., Newcastle Upon Tyne, UK). The percentages of positive caspase-3 staining cells within the cortex and hippocampus were estimated after averaging the number of cells in those areas.

### 2.7. Western Blotting

Rats were sacrificed by intraperitoneal injection of 50 mg/kg of zoletil for biochemical studies. Brain tissues were quickly removed, and the cerebral cortex and hippocampus were separated on ice. A 10% homogenate was prepared in lysis buffer, centrifuged at 12,000 (rpm) for 30 min at 4°C. Protein concentrations of samples were determined by BCA protein assay kit with BSA standards. Seventy mg of total protein was separated on 10% sodium dodecyl sulfate-polyacrylamide gels (SDS-PAGE) and transferred to polyvinylidene difluoride (PVDF) membranes. The membranes were incubated for 1 hr with 5% dry skim milk in TBST buffer at room temperature to block nonspecific binding. The membranes were then incubated with anti-caspase-3, anti-Bax, anti-Bcl-2, and anti-*β*-actin. Subsequently, the membranes were incubated with alkaline-phosphatase-conjugated secondary antibody for 1 hr at room temperature. Bands were visualized using the chromogenic substrate 5-bromo-4-chloro-3-indolyl phosphate in the presence of nitroblue tetrazolium.

### 2.8. Statistical Analysis

All data were expressed as the mean ± standard error of the mean. For single variable comparisons, Student's *t*-test was used. For multiple variable comparisons, data were analyzed by one-way ANOVA followed by Dunnett's test. *P* < 0.05 was considered significant.

## 3. Results

### 3.1. Treatment Effects on Body Weight and Plasma Glucose

Data in Table 1 show that mean body weight of the STZ-treated animals at day 21 was significantly decreased as compared to normal animals (*P* < 0.001). Treatment with GFW for 7 days increased body weight in STZ-treated rats; however, this difference was not significant (Table 1). Plasma glucose levels were significantly (*P* < 0.001) elevated in STZ-treated animals (Table 1). GFW did not alter plasma glucose levels.

### 3.2. Effects of GFW Spatial Performance Memory Deficits in Hyperglycemic Mice

**Figure 3:**
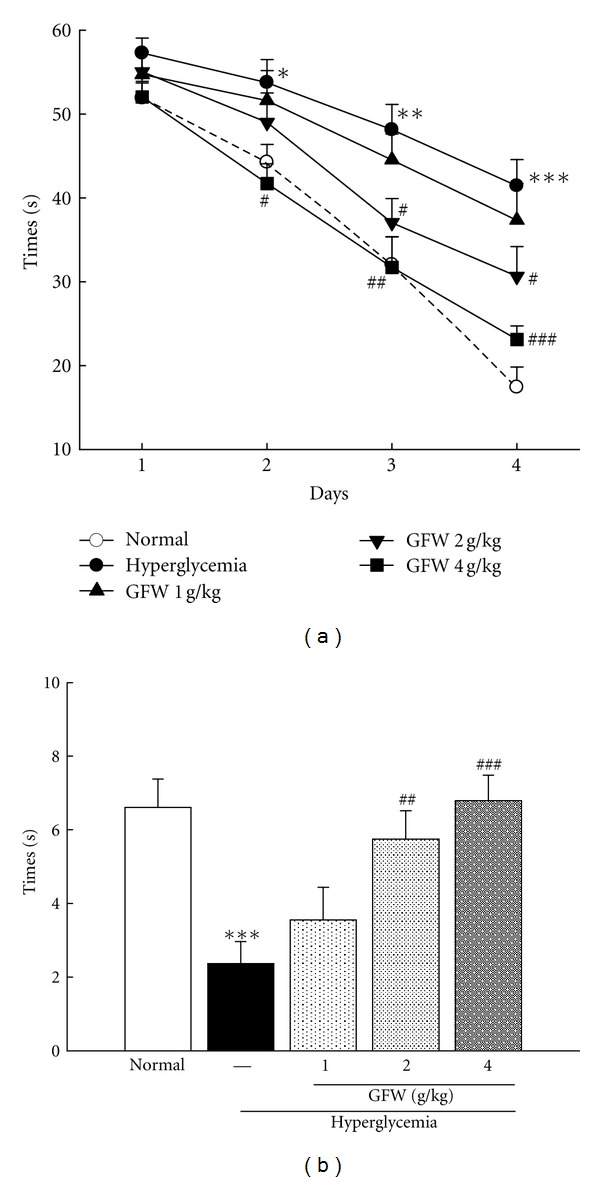
Effect of GFW (1.0, 2.0, and 4.0 g/kg) on the swimming time taken to reach the hidden platform of the Morris water maze (a) and the time spent in the target quadrant (b) in hyperglycemia mice. The performance of each mouse was tested 24 hours after the final training day in a probe trial (60 sec) during which the platform was removed. *P < 0.05, **P < 0.01, ***P < 0.001 compared to the normal group. ^#^ *P* < 0.05, ^##^ *P* < 0.01, ^###^ *P* < 0.001 compared to hyperglycemia group (*n* = 8 in each group).

Normal mice rapidly learned the location of the platform. The swimming pathway required to find the submerged platform was simplified in the normal group. In the hyperglycemic mice, a characteristic swimming behavior consisted of circling around the pool. Escape latencies in trials 1 and 2 remained unchanged throughout the testing period. In the hyperglycemic group, the swimming time was significantly increased and remained unchanged throughout the testing period. GFW at 2.0 g/kg significantly antagonized the effect in hyperglycemic mice on the escape latency on days 3 and 4 (*P* < 0.05) (Figure 3(a)). The swimming time of hyperglycemic mice treated with 4.0 g/kg GFW was significantly shorter than hyperglycemic mice.

### 3.3. Effects of GFW on Reference Memory in Hyperglycemic Mice

The time spent in the target quadrant of the hyperglycemic group was significantly reduced compared to that of the normal group and is shown in Figure 3(b) (*P* < 0.001).GFW (2.0 and 4.0 g/kg) markedly improved hyperglycemia-induced amnesia when administered before the training trial (*P* < 0.01) (Figure 3(b)).

### 3.4. TUNEL Staining

**Figure 4:**
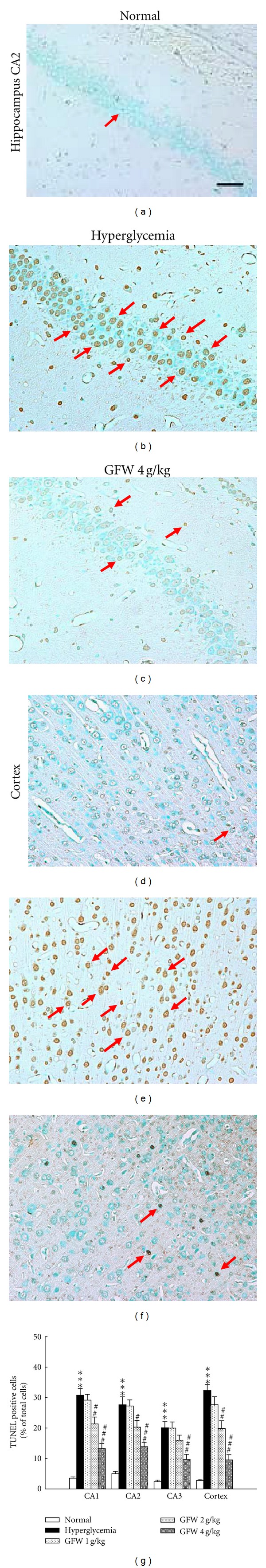
Effects of GFW on TUNEL positive cells in the cortex and hippocampus CA2 of hyperglycemic rats. (a), (d): normal; (b), (e): hyperglycemia; (c), (f): treated with GFW 4 g/kg of hyperglycemic rats. The arrow was showing the TUNEL positive cells. (g): bar chart of TUNEL positive cells in the cortex and hippocampus (CA1, CA2 and CA3) of normal rats, hyperglycemic rats, and hyperglycemic rats treated with GFW (1.0, 2.0 and 4.0 g/kg). ***P < 0.001 versus normal group. ^#^ *P* < 0.05, ^##^ *P* < 0.01, ^###^ *P* < 0.001 versus hyperglycemia group (*n* = 6 in each group). Scale bar = 5 *μ*m.

The TUNEL assay was used to determine nucleosomal DNA fragmentation. Representative micrographs of TUNEL staining in STZ-treated rats are shown in the upper panel of Figure 4. Hyperglycemic rats had significantly more TUNEL positive cells in the cortex and the hippocampus CA2 areas than the normal rats (Figures 4(b) and 4(e)). In hyperglycemic rats treated with GFW, TUNEL positive cells were reduced significantly in the cortex and the hippocampus CA2 areas compared with the hyperglycemic rats (Figures 4(c) and 4(f)). As shown in the Figure 4(g), TUNEL positive cells were significantly increased in the cortex and hippocampus CA1 to CA3 (*P* < 0.001), and treatment with GFW 2.0 g/kg could decrease hyperglycemia-induced TUNEL positive cells in the cortex and hippocampus CA1 and CA2 (*P* < 0.05). Four g/kg of GFW significantly decreased TUNEL positive cells in the cortex and hippocampus CA1, CA2, and CA3 (*P* < 0.001).

### 3.5. Cleaved Caspase-3 Expression in Hyperglycemic and GFW-Treated Hyperglycemic Rats

**Figure 5:**
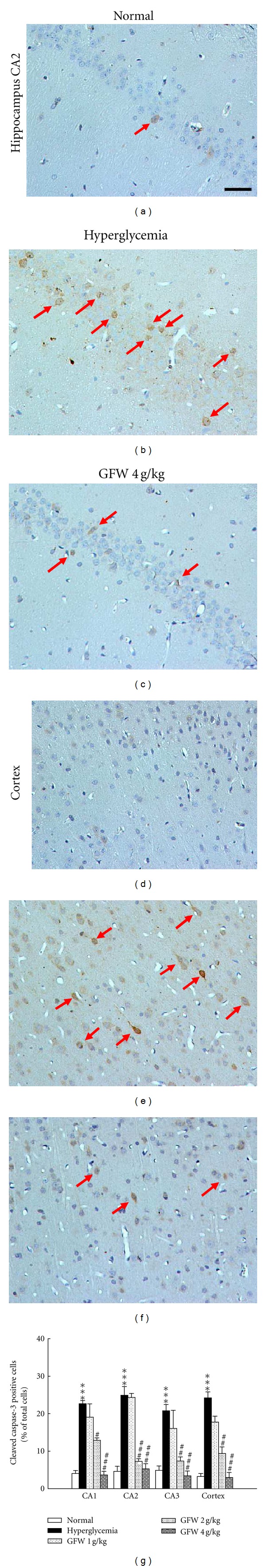
Effects of GFW on cleaved caspase-3 positive cells in the cortex and hippocampus CA2 of hyperglycemic rats. (a), (d) Normal; (b), (e) hyperglycemia; (c), (f): treated with GFW 4 g/kg of hyperglycemic rats. The arrow was showing the cleaved caspase-3 positive cells. (g) Bar chart of cleaved caspase-3 positive cells in the cortex and hippocampus (CA1, CA2, and CA3) of normal rats, hyperglycemic rats, and hyperglycemic rats treated with GFW (1.0, 2.0, and 4.0 g/kg). ***P < 0.001 versus normal group. ^#^ *P* < 0.05, ^##^ *P* < 0.01, ^###^ *P* < 0.001 versus hyperglycemia group (*n* = 6 in each group). Scale bar = 50 *μ*m.

Representative micrographs of cleaved caspase-3 positive staining in rats are seen in the upper panel of Figure 5. Normal rats had few cleaved caspase-3 positive cells in the cortex and the hippocampus CA2 areas (Figures 5(a) and 5(d)). Hyperglycemic rats had more cleaved caspase-3 positive cells than normal rats (Figures 5(b) and 5(e)). In hyperglycemic rats treated with GFW, cleaved caspase-3 positive cells were significantly reduced in the cortex and the hippocampus CA2 areas when compared with the hyperglycemic rats (Figures 5(c) and 5(f)). As shown in Figure 5(g), cleaved caspase-3 positive cells were significantly increased in the cortex and hippocampus CA1 to CA3 (*P* < 0.001), and treatment with GFW 2.0 g/kg could decrease hyperglycemia-induced cleaved caspase-3 positive cells in the cortex and hippocampus CA1, CA2 and CA3 (*P* < 0.05). Four g/kg GFW treatment significantly decreased cleaved caspase-3 positive cells in the cortex and hippocampus CA1, CA2, and CA3 (*P* < 0.001).

### 3.6. Effects of GFW on Caspase-3 Protein Abundance in the Cortex and Hippocampus

**Figure 6:**
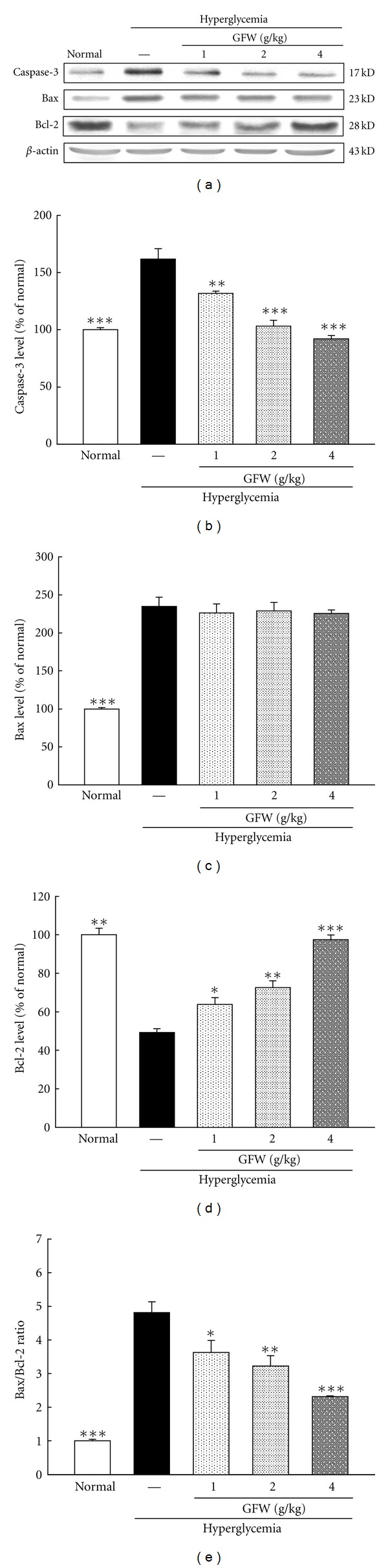
Effects of GFW (1.0, 2.0, and 4.0 g/kg) on expression of caspase-3 (b), Bax (c), Bcl-2 (d), and Bax/Bcl-2 ratio (e) in the cortex of hyperglycemic rats. Bars represent the mean ± SE from three independent experiments. *P < 0.05, **P < 0.01, ***P < 0.001 compared to hyperglycemia group (*n* = 6 in each group).

**Figure 7:**
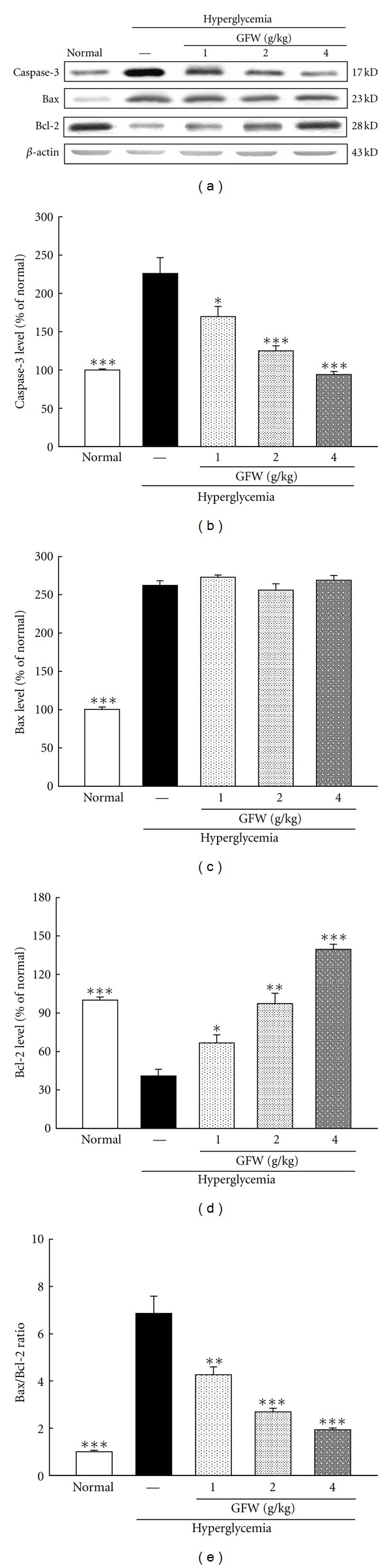
Effects of GFW (1.0, 2.0, and 4.0 g/kg) on expression of caspase-3 (b), Bax (c), Bcl-2 (d), and Bax/Bcl-2 ratio (e) in the hippocampus of hyperglycemia rats. Bars represent the mean ± SE from three independent experiments. **P < 0.01, ***P < 0.001 compared to hyperglycemia group (*n* = 6 in each group).

Caspase-3 protein levels were increased in the cortex and hippocampus of hyperglycemic rats. GFW suppressed the STZ-induced increase in caspase-3 protein abundance in the cortex and hippocampus (Figures 6(b) and 7(b)).

### 3.7. Effects of GFW on Expression of Bax and Bcl-2 in the Cortex and Hippocampus

Compared with untreated animals, STZ increased Bax protein levels but reduced Bcl-2 protein levels in the cortex and hippocampus. The Bax/Bcl-2 ratio of hyperglycemic rats increased 4.8-fold and 6.8-fold (*P* < 0.001) in the cortex and hippocampus compared with untreated animals (Figures 6(e) and 7(e)). In hyperglycemic rats, GFW did not alter Bax protein levels but significantly increased Bcl-2 protein levels resulting in a reduction in the Bax/Bcl-2 ratio which is indicative of reduced apoptosis.

## 4. Discussion

Diabetes is associated with apoptosis which could be a possible mechanism for hyperglycemia-induced neuronal cell death. The purpose of the present study was to determine if apoptotic effects of hyperglycemia induced by STZ could be reduced by treatment with GFW in the hippocampus and cerebral cortex of rats. At 3 weeks after STZ injection, rodents showed significant weight loss and elevated plasma glucose levels (Table 1). In hyperglycemic rodents treated with GFW, body weight was not significantly increased, and plasma glucose levels were not reduced. Nakagawa et al. showed that plasma glucose levels of the groups treated with GFW were significantly lower from 6 weeks than those of the untreated control (diabetes) group, but not in 3 weeks [18]. The body weight also had not significantly changed after treatment with GFW. Body weight and plasma glucose levels did not change significantly in WBN/kob rats treated with GFW [19]. In our study, GFW had no effects on body weight and plasma glucose in STZ-induced hyperglycemic animals.

The water maze is one of the most extensively used tasks in behavioral neuroscience for studying spatial learning and memory. Several studies have shown that the Morris water maze test enabled the simultaneous analysis of memory processes [20, 21]. The hippocampus and cortex are involved in acquisition, retrieval, consolidation, and storage of spatial memory. Hippocampal and cortical damage impairs Morris water maze performance [16]. Hyperglycemia produced deficits in memory performance using a Morris water maze [22, 23]. The hyperglycemic mice treated with GFW had markedly improved learning and memory as compared with untreated hyperglycemic animals.

STZ-induced hyperglycemia causes an increase in brain glucose levels. Hyperglycemia causes oxidative stress and advanced glycation end products which are associated with neuronal apoptosis [1]. Apoptosis or programmed cell death plays an important role in diabetes mellitus and chronic diseases including certain neurodegenerative diseases [2]. DNA fragmentation is characterized in apoptotic tissue and is a final, irreversible step of the apoptotic process [24]. The activation of caspases initiates the apoptotic cascade and has been divided into initiators and executioners [25]. Caspase-3 is considered as an important regulator of apoptosis, and its cleaved form is increased in neuronal apoptosis. The TUNEL assay is a commonly used marker for apoptosis-related genomic DNA fragmentation, and caspase-3 is used as another marker of apoptosis [10, 24]. In this study, TUNEL positive cells significantly increased in hyperglycemic rats, and treatment with GFW lessened those effects (Figure 4). As shown in Figures 5, 6(b), and 7(b), STZ-induced hyperglycemic rats had significantly higher cortical and hippocampal caspase-3 expression when compared with normal rats. DNA fragmentation and caspase-3 expression were significantly increased in the cortex and hippocampus of STZ-induced hyperglycemic rats compared with normal rats and are consistent with previous studies [10, 26]. GFW reduced caspase-3 activation induced by hyperglycemia. The Bcl-2 family is substantively involved in apoptosis. Bcl-2 family proteins are apoptotic regulators [27]. Hyperglycemia increased ROS in hippocampal cells resulting in elevation of intracellular calcium [9]. In the mitochondrial apoptotic pathway, reactive oxygen species and nitric oxide can change the mitochondrial membrane potential by opening mitochondrial permeability transition pore (mPTP), releasing cytochrome C and subsequent activation of caspases 9 and 3 and ultimately causing cell death. The Bcl-2 family proteins play a central role in modulating cytochrome C release and their downstream effects [9]. There are two classes of regulatory proteins in the Bcl-2 family: the antiapoptotic proteins including Bcl-2 and Bcl-xL and proapoptotic proteins including Bax and Bak [10]. Hyperglycemia-induced apoptosis is associated with increasing ratios of Bax/Bcl-2 as well as caspase-3 levels. Results from the present study showed that Bax expression was increased, and Bcl-2 protein levels were significantly reduced in the cortex and hippocampus of STZ-induced hyperglycemic rats. These changes in Bax and Bcl-2 are indicative of an apoptotic cell environment. The Bax protein levels of hyperglycemic rats treated with GFW were unchanged but Bcl-2 protein levels were significantly increased, resulting in a decrease in the Bax/Bcl-2 ratio as compared with hyperglycemic rats. GFW significantly reduced apoptosis in the cortex and hippocampus of STZ-induced hyperglycemic rats. In summary, STZ-induced hyperglycemia increased the Bax/Bcl-2 ratio, caspase-3 activation, and neuronal apoptosis in the cortex and hippocampus. Hyperglycemia associated with cognitive deficits includes learning and memory impairment in a Morris water maze test. GFW markedly improved deficits in spatial memory induced by hyperglycemia which may be due to reductions in the Bax/Bcl-2 ratio caspase-3 expression and neuronal apoptosis in the hippocampus and cortex. The hippocampus and cortex play a vital role in learning, memory, and executive function [3, 4]. Based on our results, the GFW-induced reversal in spatial memory and deficits caused by hyperglycemia may be due to decreasing apoptosis in the hippocampus and cortex.

Traditional herbal medicines have been used for thousands of years and are beneficial in prevention and treatment of many diseases, including diabetes. Greater attention is being given to such medicines due to their varied biological actions and low toxicity. This study is the first report to address the protective effects of GFW on hyperglycemia-induced memory impairment and neuronal apoptosis in rats.

## Figures and Tables

**Table 1:** Body weight and plasma glucose concentration in different days in mice and rats.

	Body weight (g)				Plasma glucose (mg/dL)			
	Mice		Rats		Mice		Rats	
	Day 0	Day 21	Day 0	Day 21	Day 0	Day 21	Day 0	Day 21
Normal	21.00 ± 0.38	35.88 ± 0.35	259.17 ± 2.71	406.67 ± 2.47	118.75 ± 4.21	120.75 ± 4.50	92.17 ± 6.51	104.33 ± 6.74
Hyperglycemia	21.38 ± 0.38	25.50 ± 0.80***	245.00 ± 3.16	206.67 ± 7.03***	119.50 ± 4.49	502.38 ± 19.20***	83.17 ± 5.28	420.50 ± 35.91***
GFW 1.0 g/kg	21.50 ± 0.38	25.75 ± 1.49***	245.83 ± 8.80	208.33 ± 8.43***	113.88 ± 2.35	507.63 ± 22.34***	83.17 ± 2.36	409.00 ± 21.60***
GFW 2.0 g/kg	21.13 ± 0.35	26.50 ± 0.76***	244.17 ± 4.55	218.33 ± 4.77***	118.88 ± 3.82	497.13 ± 16.34***	88.67 ± 4.91	400.50 ± 12.29***
GFW 4.0 g/kg	21.38 ± 0.53	27.63 ± 1.10***	251.67 ± 3.33	235.83 ± 7.57***	116.38 ± 3.20	493.38 ± 23.26***	82.50 ± 2.17	377.50 ± 19.53***

All data are expressed as the means ± SE. ***P < 0.001 compared to the normal group.
